# Lipid Profile in Adolescent Girls with Type 1 Diabetes Mellitus and Hyperandrogenemia

**DOI:** 10.1155/2016/9473158

**Published:** 2016-04-27

**Authors:** Agnieszka Zachurzok, Grazyna Deja, Aneta Gawlik, Agnieszka Drosdzol-Cop, Katarzyna Klimek, Ewa Malecka-Tendera

**Affiliations:** ^1^Department of Pediatrics and Pediatric Endocrinology, School of Medicine in Katowice, Medical University of Silesia, Medykow Street 16, 40-752 Katowice, Poland; ^2^Department of Pediatric Diabetes, School of Medicine in Katowice, Medical University of Silesia, Medykow Street 16, 40-752 Katowice, Poland; ^3^Department of Woman's Health, School of Medicine in Katowice, Medical University of Silesia, Medykow Street 12, 40-752 Katowice, Poland; ^4^Department of Instrumental Analysis, School of Pharmacy in Sosnowiec, Medical University of Silesia, Jednosci Street 8, 41-200 Sosnowiec, Poland

## Abstract

*Study Objectives*. The study aim was to evaluate whether hyperandrogenemia in adolescent girls with type 1 diabetes mellitus (T1DM) may adversely influence lipid profile.* Design and Participants*. Lipid levels in 16 diabetic girls with biochemical hyperandrogenemia (T1DM-H) aged 16.3 ± 1.2 years were compared to 38 diabetic girls with normal androgen levels (T1DM-N) aged 15.8 ± 1.2 years. 15 healthy girls served as controls (CG). In all patients, anthropometric measurements were done, and androgens and SHBG were assessed.* Results*. In T1DM-H, total cholesterol (TC) and low density cholesterol (LDL-ch) were significantly higher than in CG (196.1 ± 41.2 versus 162.7 ± 31.7 mg/dL, *p* = 0.01; 117.3 ± 33.1 versus 91.3 ± 27.8 mg/dL, *p* = 0.01, resp.). Their LDL-ch, non-high density cholesterol (non-HDL-ch) concentrations, and LDL/HDL ratio were also significantly higher than in T1DM-N (117.3 ± 33.1 versus 97.7 ± 26.7 mg/dL, *p* = 0.03; 137.3 ± 42.9 versus 113.3 ± 40.4 mg/dL, *p* = 0.04; 2.8 ± 3.7 versus 1.6 ± 0.5, *p* = 0.04, resp.). In stepwise multiple linear regression, free androgen index (FAI) and waist-to-hip ratio (WHR) were associated with TC (*R* ^2^ = 0.4, *p* < 0.0006), non-HDL-ch (*R* ^2^ = 0.4, *p* < 0.0003), and LDL-ch (*R* ^2^ = 0.4, *p* < 0.0008). Triglycerides and LDL/HDL ratio were (*R* ^2^ = 0.7, *p* < 0.0001, *R* ^2^ = 0.6, *p* < 0.0003 resp.) related to testosterone, FAI, WHR, and mean HbA1c.* Conclusion*. Lipid profile in diabetic adolescent girls is adversely influenced by the androgens level, particularly in the group with higher WHR and poorer glycemic control.

## 1. Introduction

Atherosclerosis is a common cause of morbidity and mortality in type 1 diabetes mellitus (T1DM). The mechanism involved in acceleration of cardiovascular disease (CVD) and atherosclerosis in these patients is still poorly understood. In young patients with T1DM and good glycemic control, the lipid profile is very similar to lipid profiles in the general population. However, even well-controlled diabetic youth may present subtle lipid abnormalities and serum cholesterol within the normal to borderline concentration coexistent with hyperglycemia may accelerate vascular lesions [1, 2].

Disorders of carbohydrate metabolism were described in women and adolescents with polycystic ovary syndrome (PCOS) [3, 4]. Significant positive correlation between insulin and androgen levels suggests that insulin resistance plays an important role in the pathogenesis of PCOS. In adult women with hyperandrogenism, adverse lipid profile such as increased concentration of triglycerides (TG) and non-HDL-cholesterol (non-HDL-ch) as well as decreased level of HDL-cholesterol (HDL-ch) were found. In patients with polycystic ovary syndrome (PCOS), Pirwany et al. reported increased hepatic lipase activity, enzyme responsible for the excessive production of atherogenic small dense LDL particles [3]. The activity of this enzyme is regulated by insulin resistance, estrogens, and also the androgens. Insulin resistance and hyperinsulinaemia, together with high androgens concentration, can therefore increase the risk of atherogenic lipid profile.

Increased incidence of hyperandrogenic disorders, hirsutism, and PCOS were described in adult women with T1DM [5, 6]. However, in adolescent girls clinical features of hyperandrogenism can be mild and hyperandrogenemia may be manifested only biochemically [7–9]. In these patients, high androgens concentration may contribute to dyslipidaemia, which, in coexistence with poor glycemic control, can increase the risk of cardiovascular disease. There are also data suggesting that androgen excess together with poor glycemic control may accompany development of microalbuminuria in pubertal girls [10].

The aim of the study was to establish whether hyperandrogenemia in adolescent girls with T1DM is associated with atherogenic lipid profile.

## 2. Patients and Methods

All adolescent girls attending the pediatric diabetes clinic of the University Hospital in Katowice who menstruated for more than 1 year were consecutively invited to participate in the study. Fifty-four of them were finally recruited. They were diagnosed with T1DM at least 1 year before the study entry. None of them was affected by diabetic complications, severe acne, or alopecia.

The exclusion criteria were honeymoon period, monogenic types of diabetes, type 2 diabetes mellitus, abnormal thyroid function, hyperprolactinaemia, congenital adrenal hyperplasia, consumption of medications known to influence sex steroids or SHBG in the last 3 months, chronic systemic disease, and malnutrition. All the girls participating in the study were Caucasians.

In all patients, T1DM diagnosis was confirmed (C-peptide level <0.05 nmol/L, presence of specific antibodies), and intensive insulin therapy was applied. Twenty-nine (54%) girls were treated with intermediate (NPH) or long-acting insulin analogue (glargine) and multiple daily injections (MDI) with at least three injections of rapid acting insulin analogues per day. Their mean HbA1c measured from the T1DM onset was 7.7 ± 1.7% (60.2 ± 19.1 mmol/mol) and their daily insulin requirement (DIR) was 0.9 ± 0.3 U/kg/day. Twenty-five (46%) girls were treated with continuous subcutaneous insulin infusion (CSII) by means of the insulin pump and their mean HbA1c from the time of diagnosis was 7.1 ± 1.1% (53.8 ± 11.5 mmol/mol). The difference in DIR and HbA1 level between the girls treated with MDI and CSII was statistically insignificant (*p* > 0.05). In 16 (30%) diabetic girls, increased androgens levels were found (T1DM-H); in 38 (70%) all the androgens concentrations remained within the normal range (T1DM-N). Fifteen healthy, regularly menstruating adolescent girls with no clinical signs of hyperandrogenism and with androgens level within the normal range, matched for chronological and gynecological age, served as the control group (CG).

The study was conducted according to the Declaration of Helsinki and approved by the Ethics Committee of the Medical University of Silesia. Informed consent was obtained from each subject or/and parent or guardian.

In all participants, menstrual pattern was evaluated and oligomenorrhea was defined as menstrual cycles longer than 45 days in the last six months [11]. Weight was measured with Seca scale with a precision of 100 g and height with Harpenden stadiometer to 0.1 cm. BMI and BMI *z*-score were calculated. Waist circumference (WC) and hip circumference (HC) were measured with nonelastic flexible tape and waist-to-hip ratio (WHR) was calculated. Hirsutism was evaluated by one of the two authors (Agnieszka Zachurzok and Aneta Gawlik) by means of the modified Ferriman-Gallwey score. In each girl, the transabdominal pelvic ultrasound examination was performed by the same observer (Agnieszka Drosdzol-Cop) with 5 MHz convex transducer (Siemens Acuson Antares 5.0), and volume and structure of the ovaries were evaluated. Ovaries were considered polycystic if 12 or more cysts 2–9 mm in diameter were present at least in one ovary and/or if increased ovarian volume (>10 mL) occurred. In each subject with T1DM, detailed medical history was obtained, including age at the onset of T1DM, HbA1c records from the beginning of the disease, and DIR for the last 12 months expressed as insulin units per kilograms of body weight per day. Serum concentration of lipids (total cholesterol (TC), TG, and HDL-ch), gonadotropins (LH, FSH), androstenedione (A), testosterone (T), 17-hydroxyprogesterone (17OHP), estradiol (E_2_), and sex hormone binding globulin (SHBG) were measured. Calculation of LDL-cholesterol (LDL-ch) concentration was performed according to Friedewald's equation [12]. Non-HDL-ch and LDL/HDL-cholesterol ratio (LDL/HDL) as well as free androgen index (FAI = T × 100/SHBG) and LH/FSH ratio were also calculated. Blood tests were performed during the follicular phase of menstrual cycle (3–7 days of cycle) or after 3 months of amenorrhea. Biochemical hyperandrogenemia was defined if T level exceeded 58 ng/dL, A exceeded 4.1 ng/mL, or FAI exceeded 4.5.

Plasma TC and TG levels were analyzed enzymatically, and HDL-ch concentration was measured by direct procedure using synthetic polymer and detergent (SPD procedure, Daichi). Serum levels of LH, FSH, and DHEAS were measured using chemiluminescent immunoassay by Immulite 2000 analyzer (DPC, USA). For DHEAS assay polyclonal rabbit anti-DHEAS antibody was used, with intra- and interassay variation coefficients: 7.1% and 8.2%, respectively. T and E_2_ were analyzed by electrochemiluminescence immunoassay by Cobas e 411 analyzer (Roche, Switzerland). For T assay, biotinylated monoclonal anti-T antibody (sheep) was used, with intra- and interassay variation coefficients: 5.3% and 6.3%, respectively. HbA1c was measured by high-performance liquid chromatography method, and SHBG was determined by immunoradiometric assay (Radim, Roma, Italy). 17OHP and A were analyzed by enzyme-linked immunosorbent assay (DRG Diagnostics GmbH, Germany). For 17OHP assay, polyclonal goat anti-17OHP antibody was used, with intra- and interassay variation coefficients: 5.8% and 6.8%, respectively. For A assay, polyclonal rabbit anti-A antibody was used, with intra- and interassay variation coefficients: 7.1% and 8.4%, respectively.

### 2.1. Statistical Analysis

Anthropometric data and hormonal results were compared using the Statistica 10 PL. All values were expressed as mean ± standard deviation for normal or median (interquartile range) for skewed distribution. The normality of the empirical distributions of variables was verified by Shapiro-Wilk's test and graphically by histograms. Correlation analysis was performed using Pearson correlation coefficient for normally distributed samples and Spearman correlation coefficient for nonnormally distributed data. Gamma correlation was used for nonnormal distributions with many tied ranks. Comparison between independent groups was performed using Student's *t*-test for normally distributed data and Mann-Whitney *U* test for skewed distributions. Differences between the parameters in the three groups (T1DM with normal androgen level, T1DM with hyperandrogenemia, and control group) were assessed by one-way ANOVA for normally distributed variables, followed by the post hoc test least-significant difference (LSD) for multiple comparisons. Homogeneity of variances in this analysis was verified by Levene's test. For nonnormal distributions Kruskal-Wallis test was used. To evaluate the relationship between independent anthropometric, metabolic, and hormonal variables (BMI *z*-score, WHR, HbA1c from the diagnosis of T1DM, DIR, T, A, and FAI) and dependent lipid variables (TC, TG, LDL-ch, LDL/HDL, and non-HDL-ch) in all T1DM girls, several multiple regression models were performed. The final models were adjusted by eliminating succeeding nonsignificant variables. Methods applied for statistical analysis were adequate for the sample size used in the study. *p* value <0.05 was considered statistically significant and 0.05 < *p* ≤ 0.1 was considered as a trend toward statistical significance.

## 3. Results

Clinical characteristics of the girls with T1DM with and without hyperandrogenemia and control group are shown in Table 1. There were no significant differences in BMI, BMI *z*-score, WC, and WHR between both T1DM groups and CG. The age of T1DM diagnosis, disease duration, mean HbA1c for the last 12 months, and DIR between T1DM-H and T1DM-N were not significantly different.

Hormonal results of the study and control groups are presented in Table 2. According to study criteria, androstenedione and testosterone levels as well as FAI were significantly higher in T1DM-H with respect to CG and to T1DM-N. DHEAS concentration was highest in T1DM-H and lowest in T1DM-N, the differences being statistically significant with respect to CG. There was no significant difference in 17OH progesterone between the three groups. Three (17%) T1DM-H met all the modified PCOS Rotterdam criteria, and further seven (39%) presented 2 out of 3 criteria [10]. In all these 10 girls, gynecological age exceeded 24 months.

According to the recommendations of ISPAD [13], increased level of TC (>175 mg/dL) was present in 10 (55.5%) T1DM-H and in 20 (52%) T1DM-N cases (*p* > 0.05). Non-HDL-ch above 130 mg/dL was found in 7 (39%) and 11 (29%) diabetic groups, respectively (*p* > 0.05), and LDL-ch exceeding 100 mg/dL in 11 (61%) and 18 (47%) groups, respectively (*p* > 0.05). In none of the girls from the study and control group LDL-ch exceeded 170 mg/dL.

**Figure 1:**
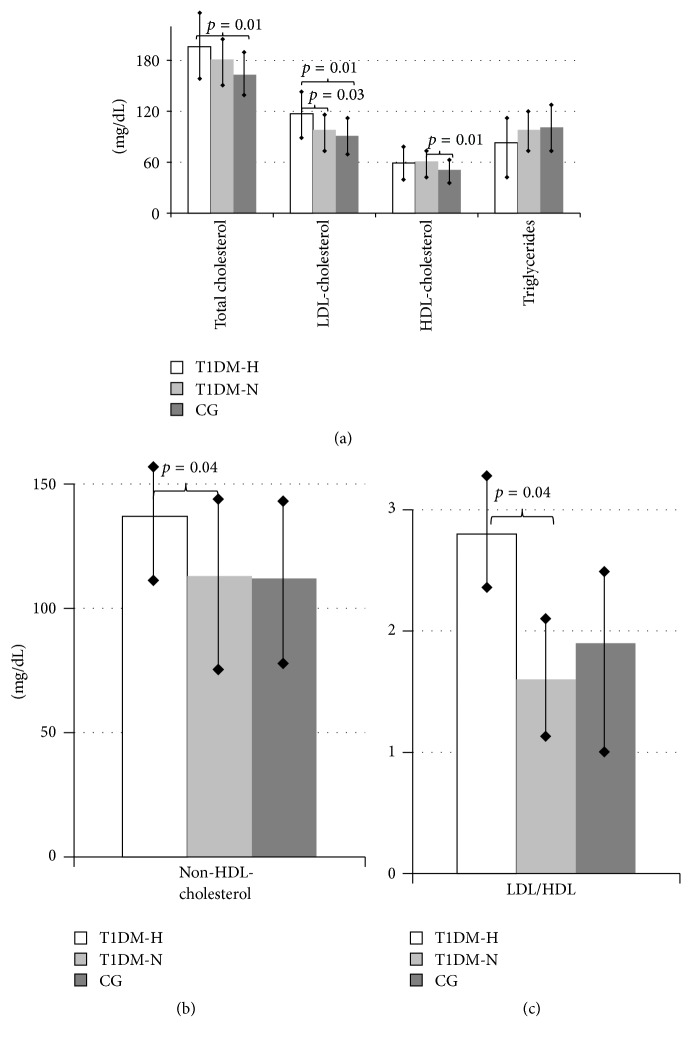
Lipid profile of adolescent girls with type 1 diabetes mellitus with (T1DM-H) and without hyperandrogenemia (T1DM-N) and healthy, regularly menstruating girls with normal androgen level (control group, CG).

Lipid profiles in the three groups of girls are presented in Figures 1(a) and 1(b). TC and LDL-ch concentration in T1DM-H were significantly higher than in CG (196.1 ± 41.2 versus 162.7 ± 31.7 mg/dL, *p* = 0.01; 117.3 ± 33.1 versus 91.3 ± 27.8 mg/dL, *p* = 0.01, resp.) Moreover, LDL-ch, non-HDL-ch concentration, and LDL/HDL ratio were also significantly higher in T1DM-H than in T1DM-N (117.3 ± 33.1 versus 97.7 ± 26.7 mg/dL, *p* = 0.03; 137.3 ± 42.9 versus 113.3 ± 40.4 mg/dL, *p* = 0.04; 2.8 ± 3.7 versus 1.6 ± 0.5, *p* = 0.04, resp.). HDL-ch concentration was similar in both diabetic groups and higher than in CG; however, significant difference was seen only between T1DM-N and CG (61.2 ± 12.4 L versus 50.6 ± 10.4 mg/dL, *p* = 0.01). There was no significant difference in TG concentration between the study and control groups.

In diabetic girls WC correlated positively with TC (*r* = 0.36, *p* = 0.03) and WHR correlated significantly with TC (*r* = 0.43, *p* = 0.007), LDL (*r* = 0.45, *p* = 0.005), and non-HDL-cholesterol (*r* = 0.42, *p* = 0.01). We found no significant relationship between lipids and BMI or BMI *z*-score. Only a tendency toward significant correlation of mean HbA1c from the onset of T1DM and TG was found (*r* = 0.28, *p* = 0.054). Correlation between lipids concentration and DIR per kg of body weight, age at T1DM diagnosis, T1DM duration, and mean HbA1c were not statistically significant. There was a significant relationship between FAI and LDL (*r* = 0.37, *p* = 0.01) and a tendency toward relationship between FAI and TC (*r* = 0.28, *p* = 0.07). Moreover, hyperandrogenemia correlated significantly with LDL (*r*_*γ*_ = 0.35, *p* = 0.01) and with LDL/HDL ratio (*r*_*γ*_ = 0.28, *p* = 0.05).

To identify anthropometric (WC, HC, and BMI *z*-score), metabolic (DIR for last 12 months, mean HbA1c from the T1DM onset), and hormonal parameters (T, A, and FAI) and their influence on lipid concentration in girls with diabetes, stepwise multiple linear regression was performed (Table 3). The regression models showed that FAI and WHR were significantly associated with TC (*R* ^2^ = 0.4, *p* < 0.001) as well as with non-HDL-ch (*R* ^2^ = 0.4, *p* < 0.001) and LDL-ch (*R* ^2^ = 0.4, *p* < 0.001). TG concentration and LDL/HDL were significantly (*R* ^2^ = 0.7, *p* < 0.001; *R* ^2^ = 0.6, *p* < 0.001, resp.) related to the mean HbA1c, WHR, T, and FAI.

To validate the influence of glycemic control on lipid profile, we divided girls with T1DM, according to ISPAD guidelines [14], into two groups: well-controlled (38 girls, mean HbA1c <7.5%, mean: 6.6 ± 0.5% (48.8 ± 5.9 mmol/mol)) and poorly controlled (16 girls, mean HbA1c ≥7.5%, mean: 9.4 ± 1.2% (79.4 ± 13.3 mmol/mol), *p* < 0.001). In poorly controlled diabetic girls BMI *z*-score and WC were higher than in well-controlled ones (1.0 (0.3–1.3) versus 0.4 (0.0–0.7), *p* = 0.02; 0.81 (0.77–0.85) cm versus 0.77 (0.75–0.79) cm, *p* = 0.04). There were no significant differences with respect to hormonal profile between the groups; however SHBG concentration was significantly higher in well-controlled group (50.6 ± 18.8 versus 37.9 ± 15.2 nmol/L, *p* = 0.04). Significantly higher TG level was found in girls with poorly controlled T1DM than in well-controlled ones (113.0 (85.0–141.0) versus 89.0 (63.0–115.0) mg/dL, *p* = 0.03). Also the tendency to higher non-HDL-ch was seen in this group (126.7 (107.9–165.3) versus 115.0 (99.6–133.1) mg/dL, *p* = 0.07). There were no significant differences with respect to other lipids between the two groups of diabetic girls.

## 4. Discussion

The relationship of hyperandrogenic disturbances with atherogenic lipid profile is documented in women with PCOS with and without T1DM [3–6, 15, 16]. However, to our knowledge, this is the first report presenting the relationship of hyperandrogenemia and lipid profile in diabetic adolescent girls.

Marked dyslipidaemia is more characteristic for type 2 diabetes, and in T1DM lipid disturbances are rather mild [17]. However in both types of diabetes, poor glycemic control increases serum triglyceride levels and decreases HDL. It can also result in a modest increase in LDL-cholesterol, which because of the elevation in triglycerides is often in the small dense subfraction. It is therefore important to optimize glycemic control in patients with diabetes because this will have secondary beneficial effects on lipid levels [1].

In our diabetic patients, mean TC exceeded ISPAD norms in both subgroups but the mean level of non-HDL-ch and LDL-ch was increased only in girls with biochemical hyperandrogenemia. On the other hand, diabetic girls in both subgroups presented HDL-ch level higher than in control group; however, only in girls without androgen excess the difference was significant. We did not assess apolipoprotein concentration, but the LDL/HDL ratio is believed to be a good ApoB/ApoA surrogate [3]. We found significantly higher LDL/HDL ratio in T1DM-H girls with respect to the other groups which can reflect more atherogenic lipid profile in these patients compared to the girls without hyperandrogenemia.

Whether endogenous hyperandrogenemia can significantly influence lipid profile remains a matter of debate. Lerchbaum et al. described an adverse metabolic phenotype in women with PCOS and high testosterone level but not in those having elevated androstenedione only [15]. Moreover, higher androstenedione/free testosterone ratio was associated with a beneficial metabolic profile. According to Wild and Bartholomew, women with PCOS had TC and non-HDL-ch levels twice as high as and HDL level significantly lower than non-PCOS controls [16]. Elevated LDL concentration was found even if women with PCOS were neither overweight nor obese. Pirwany et al. demonstrated in women with PCOS higher hepatic lipase activity that correlated not only positively with WC and WHR but also negatively with SHBG concentration [3].

In accordance with these data, we have also found the correlation between WC and WHR and atherogenic lipid profile, but we did not observe any relationship between lipid disturbances and SHBG level. However, there was a positive correlation between FAI and LDL concentration as well as between hyperandrogenemia and LDL and LDL/HDL ratio. Similar relationship was reported by Lerchbaum et al., who described in women with PCOS a positive correlation between increased free testosterone concentration and high TC and TG as well as low HDL [15].

We did not find any differences in HbA1c from the beginning of the disease as well as DIR for the last 12 months between diabetic groups with or without hyperandrogenemia. Therefore, we believe that we were able to examine the real influence of androgen excess on lipid pattern. On the other hand, we found the relationship between the glucose homeostasis and lipid profile, as in girls with poor glycemic control TG level was significantly higher.

Dabas et al. reported higher lipids in diabetic adolescents with worse glycemic control [17]. Moreover, Wild and Bartholomew revealed that in women with PCOS altered glucose-insulin homeostasis is a stronger contributor to dyslipidaemia than hyperandrogenemia or chronic high estrogen exposure [16]. These two mechanism-hyperandrogenemia, which can increase hepatic lipase activity coexisting with poor glycemic control implicated in hypertriglyceridaemia, could lead to more atherogenic lipid profile in this specific group of diabetic girls.

Another interesting finding in our research was lower DHEAS concentration in diabetic girls with no hyperandrogenemia while in hyperandrogenic girls it was comparable to the control group. Gaete et al. found that 58% of diabetic children have abnormal response to ACTH test as well as lower DHEAS concentration without any signs of hypocortisolism [18]. In type 2 diabetes, lower levels of DHEAS were also reported. Serum levels of the entire sulphoconjugated steroids were found significantly lower in well-controlled patients in comparison with poorly controlled type 2 diabetics [19]. In our diabetic subjects, there was no difference between well- and poorly controlled girls with respect to DHEAS concentration. Moreover, in diabetic hyperandrogenic girls, testosterone and androstenedione concentration as well as FAI were increased, whereas 17OHP level was similar to controls. This suggests that although the main source of androgens in girls with diabetes and hyperandrogenemia is the ovary, there may be also the upregulation of adrenal androgens production. However, this finding needs to be confirmed on a larger group of adolescents with T1DM as in the study by Meyer et al. DHEAS in diabetic adolescents was not different than that in the controls [20].

In patients with insulin-dependent diabetes, to deliver appropriate amount of insulin to the liver and achieve good metabolic control, most often supraphysiological doses of insulin are injected subcutaneously. Codner and Escobar-Morreale associated the onset of hyperandrogenism with intensive insulin therapy [21]. Hyperinsulinaemia, due mainly to cogonadotropin action, can stimulate androgens synthesis in the ovaries. In our study, girls with hyperandrogenemia had higher DIR with respect to girls with normal androgens, but the difference was statistically insignificant. However, lack of significance could have been a result of the insufficient number of patients in the groups examined.

Our study limitation is a small number of diabetic girls with elevated androgen level compared to a larger group of girls without hyperandrogenemia. Considering that girls with T1DM are less affected by clinical hyperandrogenism and the risk of PCOS development is lower than in their healthy peers, it is evident that a single center may not have a large number of patients. We also acknowledge the fact of methodological problems regarding androgens determination in young girls due to inadequate assays sensitivity to measure low concentration, interference of other steroid molecules with similar structure, and the lack of well-defined normative values. However, all the assays were performed in the same laboratory using the same kits in girls from the study and from the control group.

We have also included some girls with gynecological age below 24 months in whom irregular menses could be due to the incomplete maturation of hypothalamic-pituitary-ovarian axis. Although we were not evaluating PCOS symptoms but only biochemical hyperandrogenism in our patients, according to Rosenfield PCOS should be considered when menstrual irregularity persists for more than 1 year [22].

We conclude that hyperandrogenemia may unfavorably influence lipid profile in adolescent girls with T1DM, particularly in those with high WHR and poor glycemic control. The issue of high androgens in diabetic adolescent girls is important, not only because of adverse effect on fertility in the future life of young women, but also with respect to increased risk of cardiovascular disease and microalbuminuria. Identification of the next modifiable risk factor, in the perspective of longer life with T1DM, which is now possible with intensive diabetes care, could give this particular group of patients some practical advantages.

The study results need to be confirmed in multicenter studies with a larger number of patients.

## Figures and Tables

**Table 1:** Clinical characteristics of adolescent girls with type 1 diabetes mellitus with (T1DM-H) and without hyperandrogenemia (T1DM-N) and healthy, regularly menstruating girls with normal androgen level (control group).

	Girls with T1DM-H (*n* = 16)	Girls with T1DM-N (*n* = 38)	Control group (*n* = 15)	*p* value
Chronological age [years]	16.3 ± 1.2	15.8 ± 1.4	16.2 ± 1.2	NS
Age of menarche [years]	12.9 ± 1.2	13.3 ± 1.3	12.1 ± 1.3	NS
Gynecological age [months]	40.3 ± 18.7	30.6 ± 15.7	49.1 ± 14.4	NS
Cycle duration [days]	31.5 (28.0–36.0)	28.0 (28.0–32.0)	28.0 (28.0–32.4)	NS
BMI *z*-score	0.7 ± 0.7	0.3 ± 0.7	0.6 ± 1.4	NS
Waist-to-hip ratio	0.79 ± 0.03	1.0 ± 1.2	0.77 ± 0.04	NS
Ferriman-Gallwey score	2.1 ± 2.4	1.3 ± 2.1	2.4 ± 2.8	NS
Mean ovarian volume [ml]	4.5 ± 1.6	5.1 ± 3.4	5.7 ± 2.8	NS
Age of T1DM diagnosis [years]	7.9 ± 3.5	8.8 ± 3.5		NS
T1DM duration [years]	8.4 ± 3.3	6.9 ± 3.5		NS
Mean HbA1c from diagnosis [%]/[mmol/mol]	7.1 (6.8–8.0)/54.4 (50.8–63.9)	6.9 (6.3–7.5)/51.9 (45.4–58.5)		NS
HbA1c at study point [%]/[mmol/mol]	7.6 (6.7–9.0)/59.0 (49.7–78.9)	6.8 (6.2–7.8)/50.8 (44.3–61.2)		NS
Daily insulin requirement for the last 12 months [U/kg/day]	0.82 (0.65–0.90)	0.77 (0.66–0.98)		NS

**Table 2:** Hormonal characteristics of adolescent girls with type 1 diabetes mellitus with (T1DM-H) and without hyperandrogenemia (T1DM-N) and healthy, regularly menstruating girls without hyperandrogenemia (control group).

	Girls with T1DM-H (*n* = 16)	Girls with T1DM-N (*n* = 38)	Control group (*n* = 15)
LH [IU/L]	3.5 (1.5–6.2)	3.4 (2.0–5.1)	4.7 (3.4–6.0)
FSH [IU/L]	4.0 ± 1.5^1^	5.2 ± 2.0	5.1 ± 1.3
LH/FSH	1.1 (0.4–1.7)	0.6 (0.4–1.3)	0.9 (0.7–1.4)
Testosterone [ng/dL]	51.5 ± 23.7^1,2^	31.9 ± 13.4	36.7 ± 12.7
Androstenedione [ng/mL]	3.7 ± 1.6^1,2^	2.1 ± 0.8	2.0 ± 0.9
DHEAS [*μ*g/dL]	201.9 ± 49.9^1^	151.5 ± 63.3^3^	213.2 ± 85.0
17OH-progesterone [ng/mL]	1.1 (1.0–1.4)	1.1 (0.8–1.4)	1.1 (0.7–1.2)
Estradiol [pmol/L]	90.7 (74.7–126.5)	116.0 (88.8–158.0)	107.0 (82.2–124.0)
SHBG [nmol/L]	38.2 ± 19.8	50.4 ± 17.2	52.2 ± 24.0
FAI	4.8 ± 2.5^1,2^	2.2 ± 0.9	2.8 ± 1.3

^1^T1DM-H versus T1DM-N: *p* < 0.03; ^2^T1DM-H versus CG: *p* < 0.01; ^3^T1DM-N versus CG: *p* = 0.004.

SHBG: sex hormone binding globulin; FAI: free androgen index.

**Table 3:** Stepwise multiple linear regression models for lipids concentrations with respect to independent anthropometric, metabolic, and hormonal variables.

	*B*	SE (*B*)	*β*	*R* ^2^	*p*
Total cholesterol					
WHR	301.7	116.7	0.4	0.04	0.02
FAI	9.4	3.1	0.4	0.04	0.006
Triglycerides					
Mean HbA1c from the diagnosis of T1DM	21.9	5.3	0.5	0.2	<0.001
WHR	407.5	116.3	0.4	0.06	0.002
Testosterone	−1.6	0.5	−0.4	0.2	0.002
FAI	10.9	3.7	0.4	0.3	0.007
LDL-cholesterol					
WHR	266.3	94.2	0.4	0.04	0.008
FAI	6.5	2.5	0.4	0.04	0.02
Non-HDL-cholesterol					
WHR	340.8	112.4	0.4	0.04	0.005
FAI	8.9	3.0	0.4	0.04	0.006
LDL/HDL					
Mean HbA1c from the diagnosis of T1DM	0.2	0.1	0.3	0.3	0.04
WHR	5.8	1.8	0.4	0.2	0.004
Testosterone	−0.02	0.0	−0.4	0.4	0.008
Androstenedione	0.3	0.1	0.7	0.7	0.007
FAI	0.2	0.1	0.7	0.7	0.007

WHR: waist-to-hip ratio; FAI: free androgen index.

## References

[1] Guy J., Ogden L., Wadwa R. P. (2009). Lipid and lipoprotein profiles in youth with and without type 1 diabetes: the SEARCH for diabetes in youth case-control study. *Diabetes Care*.

[2] Machnica L., Deja G., Polanska J., Jarosz-Chobot P. (2014). Blood pressure disturbances and endothelial dysfunction markers in children and adolescents with type 1 diabetes. *Atherosclerosis*.

[3] Pirwany I. R., Fleming R., Greer I. A., Packard C. J., Sattar N. (2001). Lipids and lipoprotein subfractions in women with PCOS: relationship to metabolic and endocrine parameters. *Clinical Endocrinology*.

[4] Wild R. A. (2012). Dyslipidemia in PCOS. *Steroids*.

[5] Codner E., Soto N., Lopez P. (2006). Diagnostic criteria for polycystic ovary syndrome and ovarian morphology in women with type 1 diabetes mellitus. *Journal of Clinical Endocrinology and Metabolism*.

[6] Escobar-Morreale H. F., Roldán B., Barrio R. (2000). High prevalence of the polycystic ovary syndrome and hirsutism in women with type 1 diabetes mellitus. *Journal of Clinical Endocrinology and Metabolism*.

[7] Zachurzok A., Deja G., Gawlik A., Drosdzol-Cop A., Małecka-Tendera E. (2013). Hyperandrogenism in adolescent girls with type 1 diabetes mellitus treated with intensive and continuous subcutaneous insulin therapy. *Endokrynologia Polska*.

[8] Bouzas I. C., Cader S. A., Leão L., Kuschnir M. C., Braga C. (2014). Menstrual cycle alterations during adolescence: early expression of metabolic syndrome and polycystic ovary syndrome. *Journal of Pediatric and Adolescent Gynecology*.

[9] Witchel S. F., Oberfield S., Rosenfield R. L. (2015). The diagnosis of polycystic ovary syndrome during adolescence. *Hormone Research in Paediatrics*.

[10] Amin R., Schultz C., Ong K. (2003). Low IGF-I and elevated testosterone during puberty in subjects with type 1 diabetes developing microalbuminuria in comparison to normoalbuminuric control subjects: the Oxford Regional Prospective study. *Diabetes Care*.

[11] Carmina E., Oberfield S. E., Lobo R. A. (2010). The diagnosis of polycystic ovary syndrome in adolescents. *American Journal of Obstetrics and Gynecology*.

[12] Friedewald W. T., Levy R. I., Fredrickson D. S. (1972). Estimation of the concentration of low-density lipoprotein cholesterol in plasma, without use of the preparative ultracentrifuge. *Clinical Chemistry*.

[13] Donaghue K. C., Wadwa R. P., Dimeglio L. A. (2014). ISPAD clinical practice consensus guidelines 2014 compendium: microvascular and macrovascular complications in children and adolescents. *Pediatric Diabetes*.

[14] Rewers M. J., Pillay K., de Beaufort C. (2014). ISPAD Clinical Practice Consensus Guidelines 2014 Compendium: assessment and monitoring of glycemic control in children and adolescents with diabete. *Pediatric Diabetes*.

[15] Lerchbaum E., Schwetz V., Rabe T., Giuliani A., Obermayer-Pietsch B. (2014). Hyperandrogenemia in polycystic ovary syndrome: exploration of the role of free testosterone and androstenedione in metabolic phenotype. *PLoS ONE*.

[16] Wild R. A., Bartholomew M. J. (1988). The influence of body weight on lipoprotein lipids in patients with polycystic ovary syndrome. *American Journal of Obstetrics and Gynecology*.

[17] Dabas A., Yadav S., Gupta V. K. (2014). Lipid profile and correlation to cardiac risk factors and cardiovascular function in type 1 adolescent diabetics from a developing country. *International Journal of Pediatrics*.

[18] Gaete X., Iñiguez G., Linares J., Avila A., Mericq V. (2013). Cortisol hyporesponsiveness to the low dose ACTH test is a frequent finding in a pediatric population with type 1 diabetes mellitus. *Pediatric Diabetes*.

[19] Tagawa N., Ohta M., Nakamura N. (2002). Serum concentrations of delta 5-3 *β*-hydroxysteroids in type 2 diabetes mellitus. *Biological and Pharmaceutical Bulletin*.

[20] Meyer K., Deutscher J., Anil M., Berthold A., Bartsch M., Kiess W. (2000). Serum androgen levels in adolescents with type 1 diabetes: relationship to pubertal stage and metabolic control. *Journal of Endocrinological Investigation*.

[21] Codner E., Escobar-Morreale H. F. (2007). Hyperandrogenism and polycystic ovary syndrome in women with type 1 diabetes mellitus. *Journal of Clinical Endocrinology and Metabolism*.

[22] Rosenfield R. L. (2013). Clinical review: adolescent anovulation-maturational mechanisms and implications. *Journal of Clinical Endocrinology and Metabolism*.

